# Urokinase-type plasminogen activator receptor promotes proliferation and invasion with reduced cisplatin sensitivity in malignant mesothelioma

**DOI:** 10.18632/oncotarget.11829

**Published:** 2016-09-02

**Authors:** Shenqi Wang, Li Jiang, Yipeng Han, Shan Hwu Chew, Yuuki Ohara, Shinya Akatsuka, Liang Weng, Koji Kawaguchi, Takayuki Fukui, Yoshitaka Sekido, Kohei Yokoi, Shinya Toyokuni

**Affiliations:** ^1^ Department of Pathology and Biological Responses, Nagoya University Graduate School of Medicine, Nagoya, 466–8550, Japan; ^2^ Department of Tumor Pathology, Nagoya University Graduate School of Medicine, Nagoya, 466–8550, Japan; ^3^ Department of Thoracic Surgery, Nagoya University Graduate School of Medicine, Nagoya, 466–8550, Japan; ^4^ Department of Cancer Genetics, Nagoya University Graduate School of Medicine, Nagoya, 466–8550, Japan; ^5^ Division of Molecular Oncology, Aichi Cancer Center Research Institute, Nagoya, 464–8681, Japan

**Keywords:** malignant mesothelioma, uPAR, cisplatin, AKT signaling pathway

## Abstract

Malignant mesothelioma (MM) is a rare neoplasm associated with asbestos exposure. The prognosis of MM is poor because it is aggressive and highly resistant to chemotherapy. Using a rat model of asbestos-induced MM, we found elevated urokinase-type plasminogen activator receptor (*uPAR; Plaur*) expression in rat tissues, which was associated with poor prognosis. The proliferation, migration and invasion of MM cells were suppressed by *uPAR* knockdown and increased by overexpression experiments, irrespective of urokinase-type plasminogen activator (*uPA; Plau*) levels. More importantly, we found that *uPAR* expression is associated with sensitivity to cisplatin in MM through the PI3K/AKT pathway, which was demonstrated with specific inhibitors, LY294002 and Akti-1/2. *uPAR* knockdown significantly increased sensitivity to cisplatin whereas its overexpression significantly decreased cisplatin sensitivity. Furthermore, sera and tissues from MM patients showed significantly high uPAR levels, which suggested the pathogenic role of uPAR in the tumor biology of human MM. In conclusion, our findings indicate that uPAR levels are associated with malignant characteristics and cisplatin sensitivity of MM. In addition to the potential use of uPAR as a prognostic marker, the combination of uPAR abrogation and cisplatin may reveal a promising therapeutic approach for MM.

## INTRODUCTION

Malignant mesothelioma (MM) arises from the serosal mesothelial cells of somatic cavities and is an aggressive neoplasm [1–4]. Exposure to asbestos is the primary cause of MM [5]. The incidence of MM is increasing worldwide, especially in developing countries [6, 7]. Chemotherapy, surgery and radiation are rarely curative for MM. Among these treatments, chemotherapy is a relatively effective treatment for MM, and combining cisplatin with pemetrexed improves patient survival. However, the median progression-free survival time is only 6.0 months, and the median overall survival time is 14.7 months [8]. Therefore, it is critical to identify key molecules for the early diagnosis of MM and development of novel therapies.

Urokinase-type plasminogen activator receptor (*uPAR; Plaur*), also known as CD87, is a glycosyl-phosphatidylinositol (GPI)-anchored membrane protein [9]. While *uPAR* is normally expressed in various parts of the body, such as the colon, kidney, bronchus and bone marrow (www.proteinatlas.org), its expression increases during myeloid/monocytic differentiation [10], wound healing in keratinocytes [11] and the progression of various neoplasms [12]. It was originally identified as a cell-surface binding site for urokinase-type plasminogen activator (*uPA; Plau*). Although uPAR does not contain any transmembrane or cytosolic domains, it is anchored to the plasma membrane through a GPI moiety, which is simultaneously added with the posttranscriptional removal of a COOH terminal signal sequence [13]. Recent studies have suggested that uPAR can also act as a signaling receptor in cooperation with transmembrane receptors like vitronectin/integrin to activate major intracellular signaling pathways, such as the phosphatidyl inositide 3-kinase (PI3K)/AKT pathway [14, 15].

uPAR has been demonstrated as a component of the main systems involved in the growth, metastasis and angiogenesis of several solid and hematologic malignancies [16, 17]. Moreover, elevated plasma levels of its cleaved form, called soluble uPAR, are frequently associated with poor prognosis in breast and colorectal cancers [18]. However, no studies have examined the serum or tissue uPAR levels in asbestos-induced MM and their relation to the malignancy and sensitivity to chemotherapy drugs, except for a phenotype investigation in an orthopedic mouse transplant model [19]. Here, for the first time, we measured and modulated *uPAR* expression in asbestos-induced MM tissues and cells to determine downstream signaling alterations and its impact on chemotherapy. The implications of these observed effects on the treatment and prognosis of MM are also discussed.

## RESULTS

### uPAR increase in asbestos-induced rat MM and its association with prognosis

**Figure 1:**
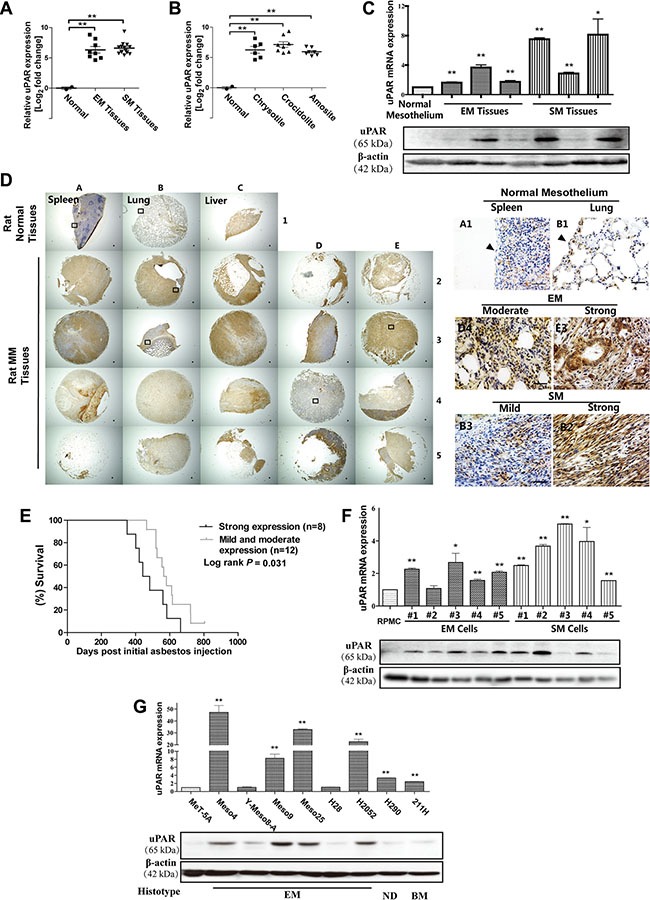
*uPAR (Plaur)* overexpression in rat asbestos-induced malignant mesothelioma (MM) and rat/human MM cell lines in association with prognosis Microarray *uPAR* expression for (**A**) histological subtype and (**B**) asbestos fiber. (**C**) *uPAR* expression in rat MM tissues with quantitative RT-PCR and Western blot analysis. (**D**) uPAR immunostaining in rat tissue array with spleen/lung/liver (rat normal tissues from left to right) surface lining mesothelial cells as control (arrow, bar = 50 μm). (**E**) Strong uPAR expression in rat MM was associated with poorer survival. (**F**, **G**) *uPAR* expression in rat and human MM cell lines with quantitative RT-PCR and Western blot analysis. MM, malignant mesothelioma; EM, epithelioid subtype mesothelioma; SM, sarcomatoid subtype mesothelioma; BM, biphasic subtype mesothelioma; ND, mesothelioma of not determined subtype (means ± SEM; representative of three independent assays; *P < 0.05, **P < 0.01). See text for details.

Based on our previous data of asbestos-induced MM (GEO Accession No. GSE48298) in rats, both histological subtypes of MM, i.e., the epithelioid (EM) and sarcomatoid (SM) subtypes, showed approximately 6–7-fold increase in *uPAR* expression compared with scraped normal mesothelial cells (Figure 1A). Similar results were observed for MM induced by 3 different types of asbestos (Figure 1B). We confirmed the results with quantitative RT-PCR, Western blot (Figure 1C, Supplementary Figure S1A) and immunohistochemistry analyses (Figure 1D). In rat MM tissue array analysis, the majority of *uPAR* expression was localized in the cytoplasm and plasma membrane in rat MM tissue cores, and *uPAR* expression in normal spleen and lung mesothelium was almost negative. Then, we semiquantified the staining density for each MM tissue core using the H-score formulation, as previously described [20]; out of 20 rat MM cores, there were 2 cases of mild, 10 cases of moderate and 8 cases of strong immunostaining. A survival analysis was performed between the combined mild and moderate expression groups and the strong expression group according to the H-score threshold of 200. The results showed that strong *uPAR* expression in rat MM was associated with significantly shorter survival during carcinogenesis (Figure 1E).

### Rat/human MM cell lines

The rat MM cell lines showed results that were consistent with those of the corresponding original tumors. The human MM cells consisted of 7 EM, 1 biphasic subtype (BM) and 1 not determined subtype (ND), which revealed similar elevated *uPAR* expression, except for the Y-Meso-8A and H28 cells, compared to an immortalized rat peritoneal mesothelial cell line (RPMC) and a transformed normal human mesothelial cell line (MeT-5A) (Figure 1F, 1G, Supplementary Figure S1B, S1C).

### Knockdown of *uPAR* suppresses the proliferation, migration and invasion of rat MM cells

**Figure 2:**
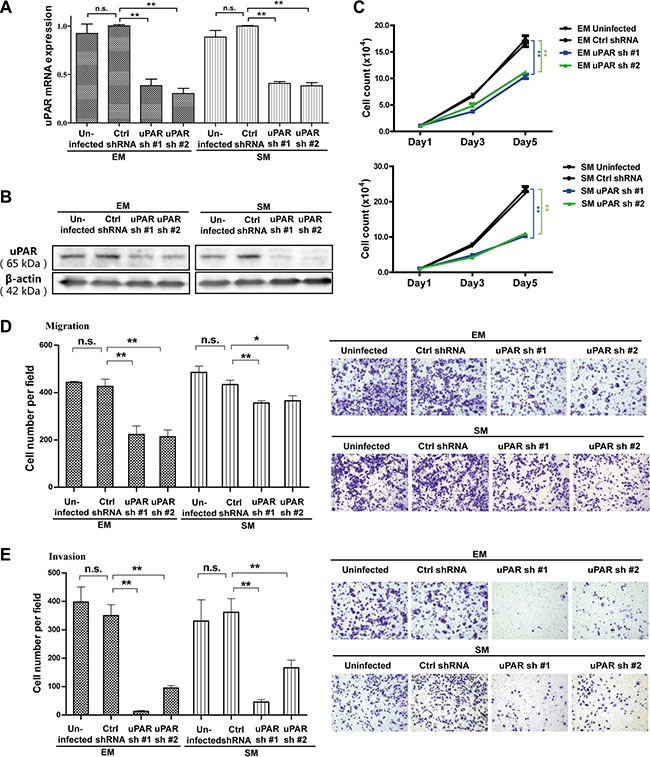
Inhibition of proliferation, migration and invasion with *uPAR* knockdown in rat EM and SM cells The knockdown efficiency of two *sh*RNA sequences transduced by lentivirus, targeting *uPAR* in EM and SM cells, as determined by (**A**) quantitative RT-PCR and (**B**) Western blot. Suppressed proliferation, migration and invasion of rat MM cells after *uPAR* knockdown were observed in EM and SM cells by (**C**) cell counting, (**D**) transwell migration and (**E**) transwell invasion analyses. EM, epithelioid subtype mesothelioma; SM, sarcomatoid subtype mesothelioma (means ± SEM; *P < 0.05, **P < 0.01; m.s., not significant). See text for details.

To explore the potential role of uPAR in MM cell growth, motility and invasion, we stably transfected either rat *uPAR*-targeted *sh*RNAs (*uPAR sh*RNA #1 and #2) or a luciferase-targeted *sh*RNA as a control in EM and SM cells via lentiviral infection. Validation of *uPAR* knockdown was evaluated by quantitative RT-PCR and Western blot analyses. Meanwhile, compared with uninfected MM cells, viral infection did not obviously change the *uPAR* expression level in control *sh*RNA-infected MM cells (Figure 2A, 2B, Supplementary Figure S1D). In both the EM and SM cell lines, stable *uPAR* knockdown resulted in significantly suppressed proliferation, as determined by cell counting with the trypan blue exclusion method (Figure 2C). Furthermore, the migratory and invasive properties of the rat MM cells were also inhibited, as determined by transwell assays (Figure 2D, 2E).

### Effect of *uPAR* knockdown is independent of uPA in rat MM cells

**Figure 3:**
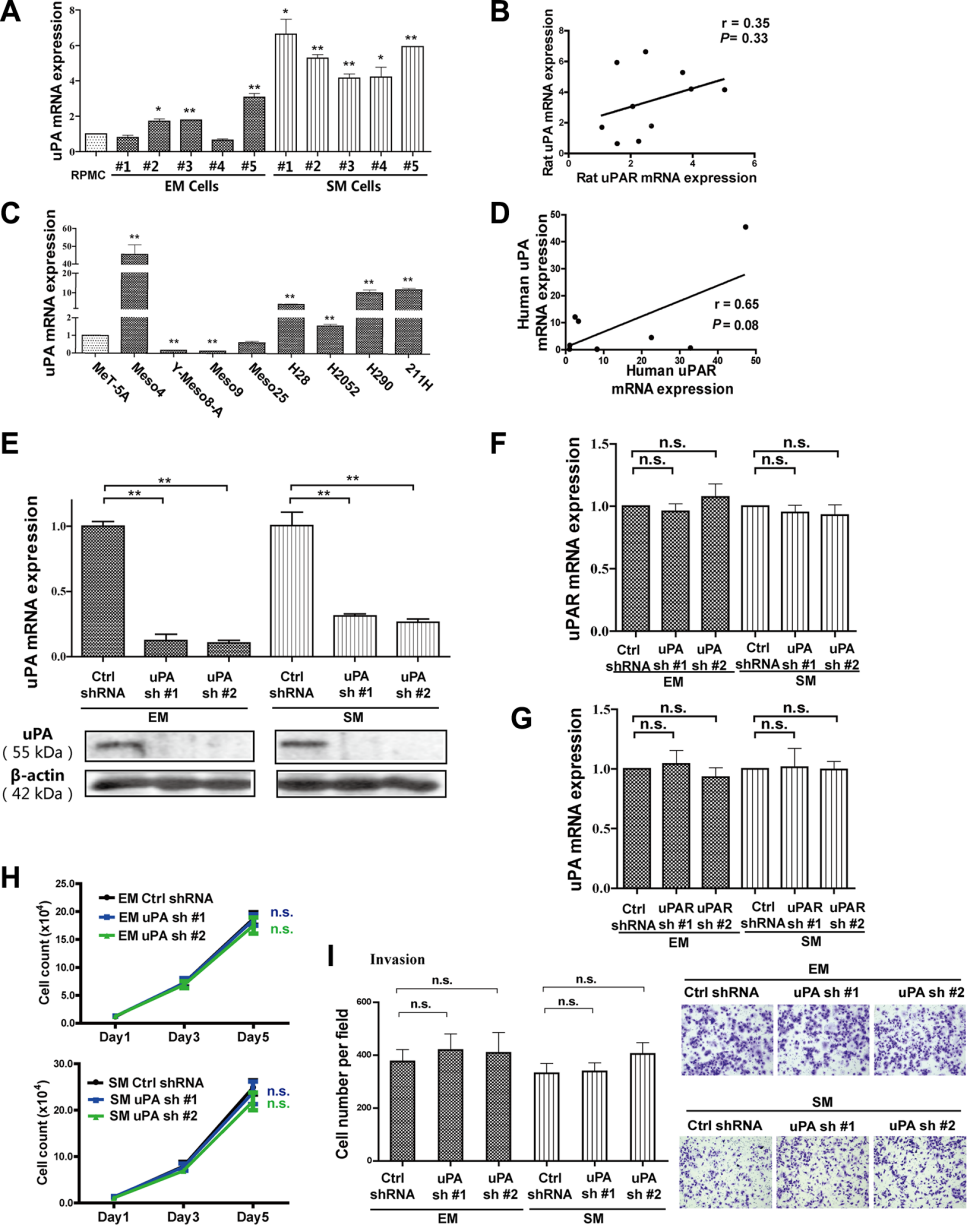
Mutual irrelevance between *uPA* and *uPAR* expression in rat/human MM cells and no effect on proliferation and invasion with *uPA* knockdown in rat EM and SM cells (**A**, **C**) *uPA* expression in rat/human MM cells with quantitative RT-PCR and (**B**, **D**) no significant correlation between *uPA* and *uPAR* expression. Knockdown efficiency of two *sh*RNA sequences transduced by lentivirus, targeting *uPA* in EM and SM cells, determined by (**E**) quantitative RT-PCR and Western blot analyses. There are no significant changes in (**F**) *uPAR* expression after *uPA* knockdown and (**G**) *uPA* expression after *uPAR* knockdown with quantitative RT-PCR. There is no obvious suppression of proliferation and invasion of rat MM cells after *uPA* knockdown, as observed in EM and SM cells by (**H**) cell counting and (**I**) transwell invasion analyses. EM, epithelioid subtype mesothelioma; SM, sarcomatoid subtype mesothelioma (means ± SEM; *P < 0.05, **P < 0.01; m.s., not significant). See text for details.

To further determine whether *uPA*, as a *uPAR* ligand involved in MM cell growth motility and invasion, the relationships of *uPA* and *uPAR* expression in rat and human MM cells were investigated by quantitative RT-PCR. There was no significant correlation between *uPA* and *uPAR* in MM cells (Figure 3A–3D). We also used rat *uPA*-targeted *sh*RNAs (*uPA sh*RNA #1 and #2) in EM and SM cells. Validation of *uPA* knockdown was evaluated by quantitative RT-PCR and Western blot analyses (Figure 3E, Supplementary Figure S1E). However, our results revealed no mutual influence between *uPA* and *uPAR* by *uPA* or *uPAR* knockdown experiments in rat MM cells through quantitative RT-PCR analysis (Figure 3F, 3G). Moreover, proliferation and invasion of rat MM cells were not significantly inhibited by *uPA* knockdown (Figure 3H, 3I).

### *uPAR* overexpression stimulates the proliferation, migration and invasion in a human MM cell line

**Figure 4:**
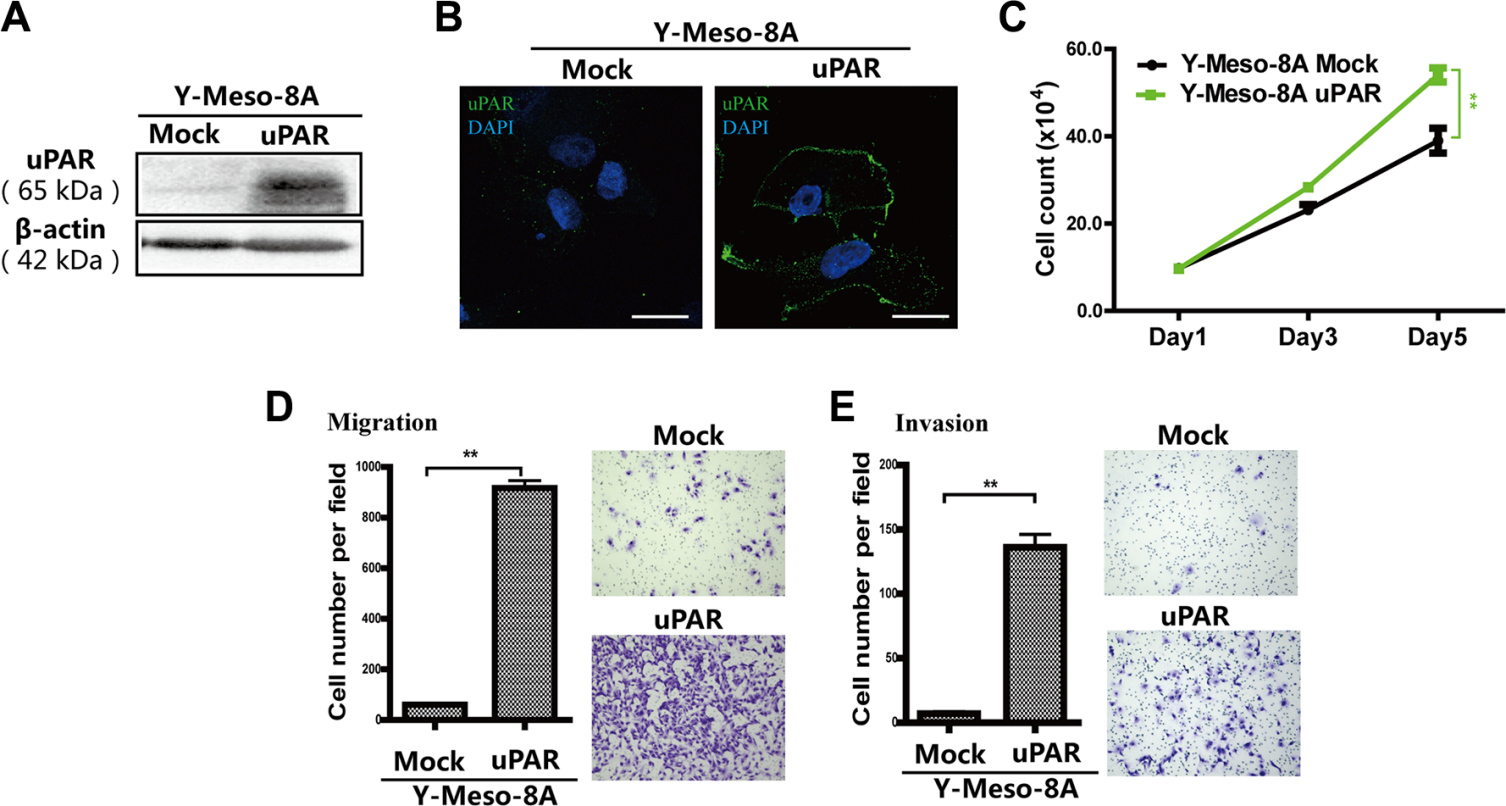
Enhanced proliferation, migration and invasion after *uPAR* overexpression in Y-Meso-8A cells *uPAR* overexpression in Y-Meso-8A cells was determined by (**A**) Western blot and (**B**) immunofluorescence analyses (bar = 20 μm). Enhanced proliferation, migration and invasion after *uPAR* overexpression were observed in Y-Meso-8A cells by (**C**) cell counting, (**D**) transwell migration and (**E**) transwell invasion analyses (means ± SEM; *P < 0.05, **P < 0.01). See text for details.

Conversely, we overexpressed *uPAR* in a human MM cell line with low *uPAR* expression, Y-Meso-8A, using a human *uPAR* expression vector. Immunofluorescence analysis indicated that overexpressed uPAR was mainly localized on the cell membrane (Figure 4A, 4B, Supplementary Figure S1F). *uPAR* overexpression stimulated proliferation, migration and invasion in Y-Meso-8A cells (Figure 4C–4E).

### Effect of *uPAR* expression on the *AKT/mTOR* signaling pathway

**Figure 5:**
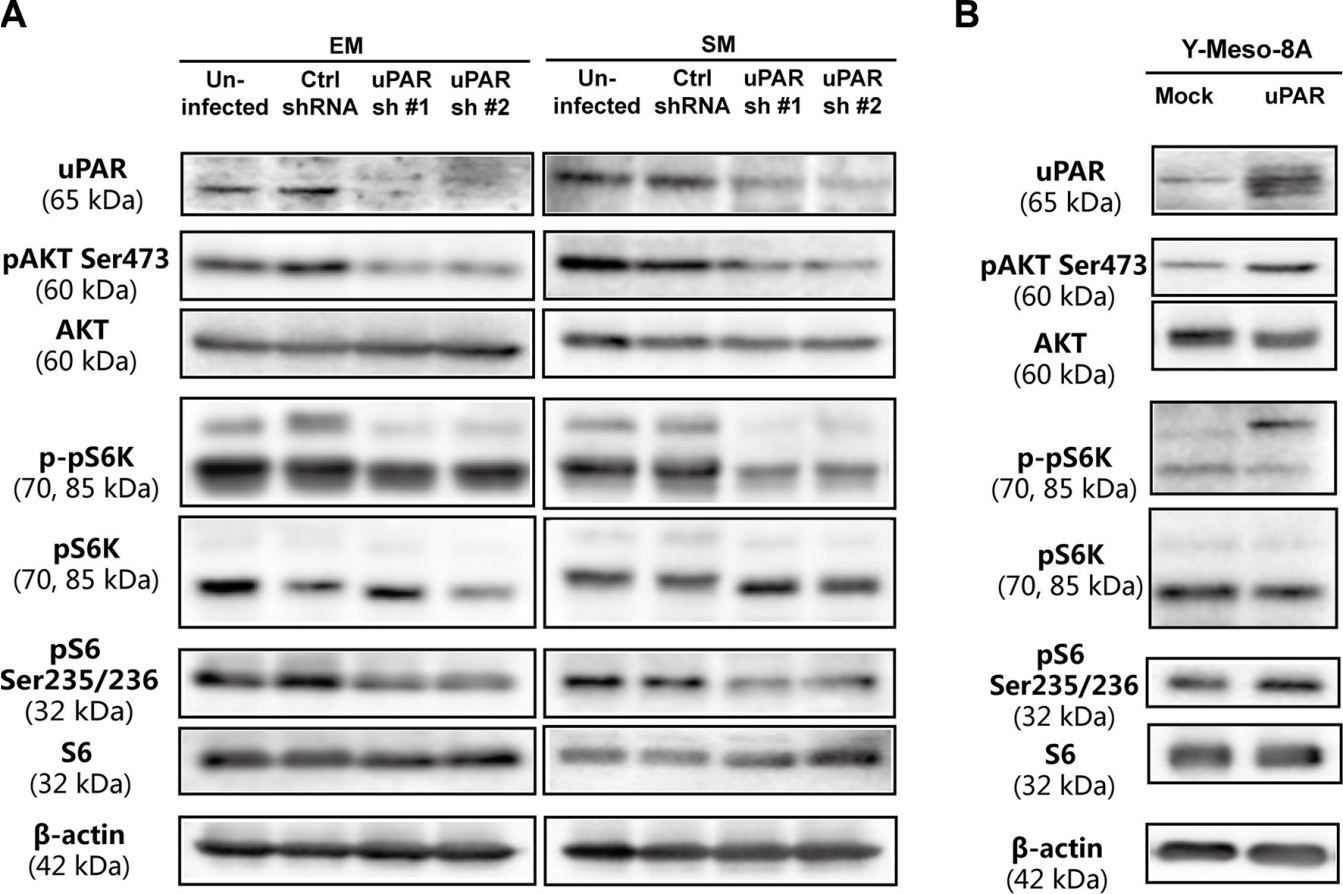
Determination of AKT activity and its downstream signalling pathway (**A**) Downregulated AKT and downstream mTOR signaling pathway after *uPAR* knockdown in EM and SM cells. (**B**) Upregulated AKT and downstream mTOR signaling pathway after *uPAR* overexpression in Y-Meso-8A cells. Refer to Figure 2 and 3 for *uPAR* knockdown and overexpression.

To further evaluate alterations in intracellular signaling after modulating *uPAR* expression, we evaluated *AKT*, which was suggested to be frequently activated in MM cells (Supplementary Figure S2A, S2B) and downstream proteins, *pS6K* and *S6*, which are important components of the *mTOR* pathways [21]. We observed that AKT/mTOR activity was closely associated with *uPAR* expression, as demonstrated through *uPAR* knockdown and overexpression in MM cells (Figure 5A, 5B, Supplementary Figure S1G, S1H). The extracellular signal-regulated kinase (ERK) is also reported to be regulated by *uPAR* expression [22]. Nevertheless, Western blot analysis showed that *uPAR* expression exerted no obvious effect on ERK in MM cells (Supplementary Figure S3A, S3B).

### Knockdown of *uPAR* decreases cell growth and the AKT signaling pathway *in vivo*

**Figure 6:**
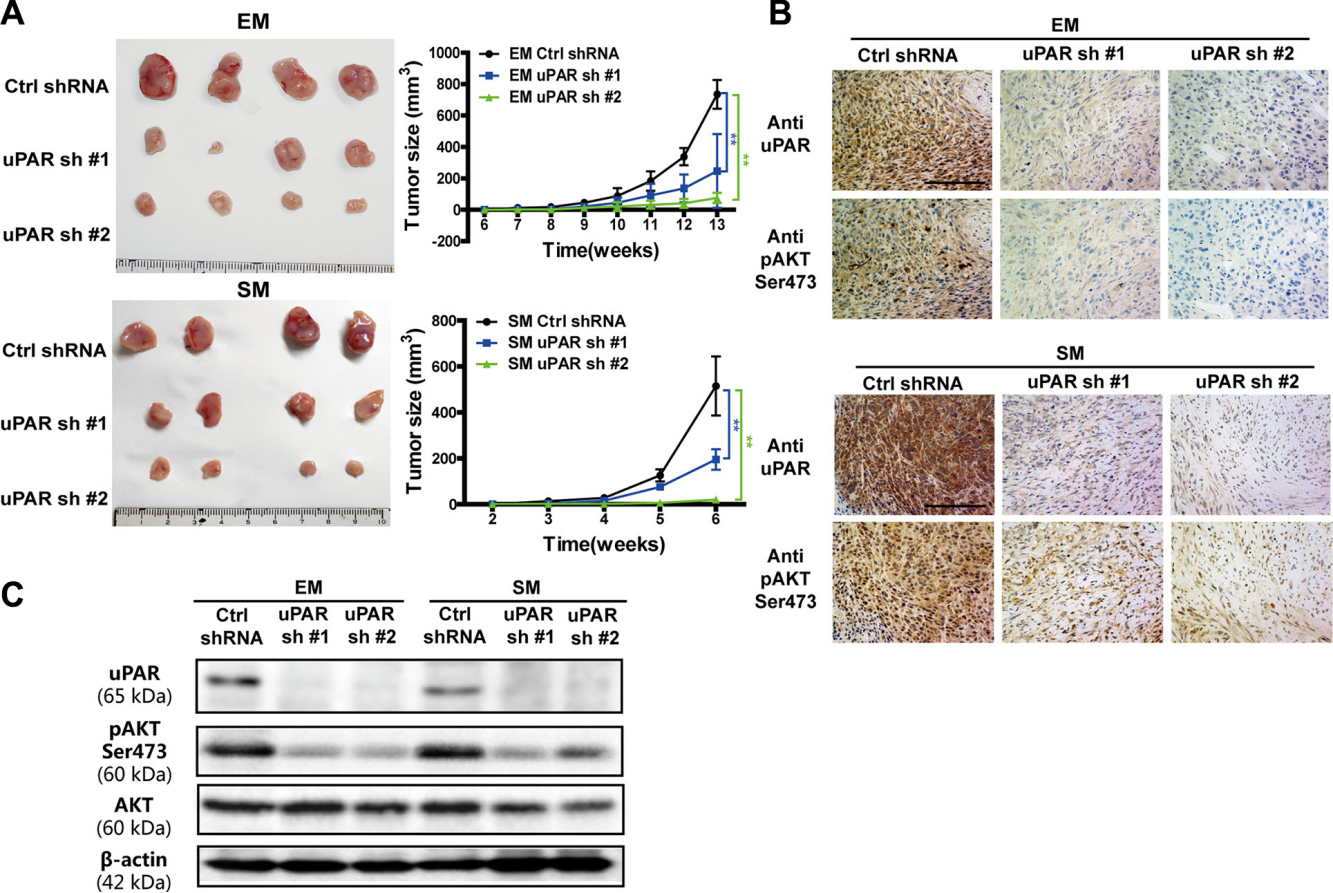
Inhibited growth of MM tumors *in vivo* with *uPAR* knock down (**A**) The growth suppression *in vivo* upon *uPAR* knockdown in EM and SM cells in nude mice (*n* = 4 per group). uPAR and pAKT expression in xenograft tumors with (**B**) immunochemistry and (**C**) Western blot analyses. EM, epithelioid subtype mesothelioma; SM, sarcomatoid subtype mesothelioma (means ± SD; *P < 0.05, **P < 0.01).

To further evaluate whether *uPAR* is crucial for the growth of MM cells and has an effect on the AKT signaling *in vivo*, we utilized a nude mice xenograft model. The tumor growth curves derived from the xenograft experiments indicated that *uPAR* knockdown significantly impeded MM cell growth in nude mice (Figure 6A). Moreover, AKT showed decreased activity after *uPAR* knockdown in MM tumors by immunohistochemistry and Western blot analyses (Figure 6B, 6C, Supplementary Figure S1I).

### Cisplatin sensitivity in rat MM cells is proportionally associated with *uPAR* expression

**Figure 7:**
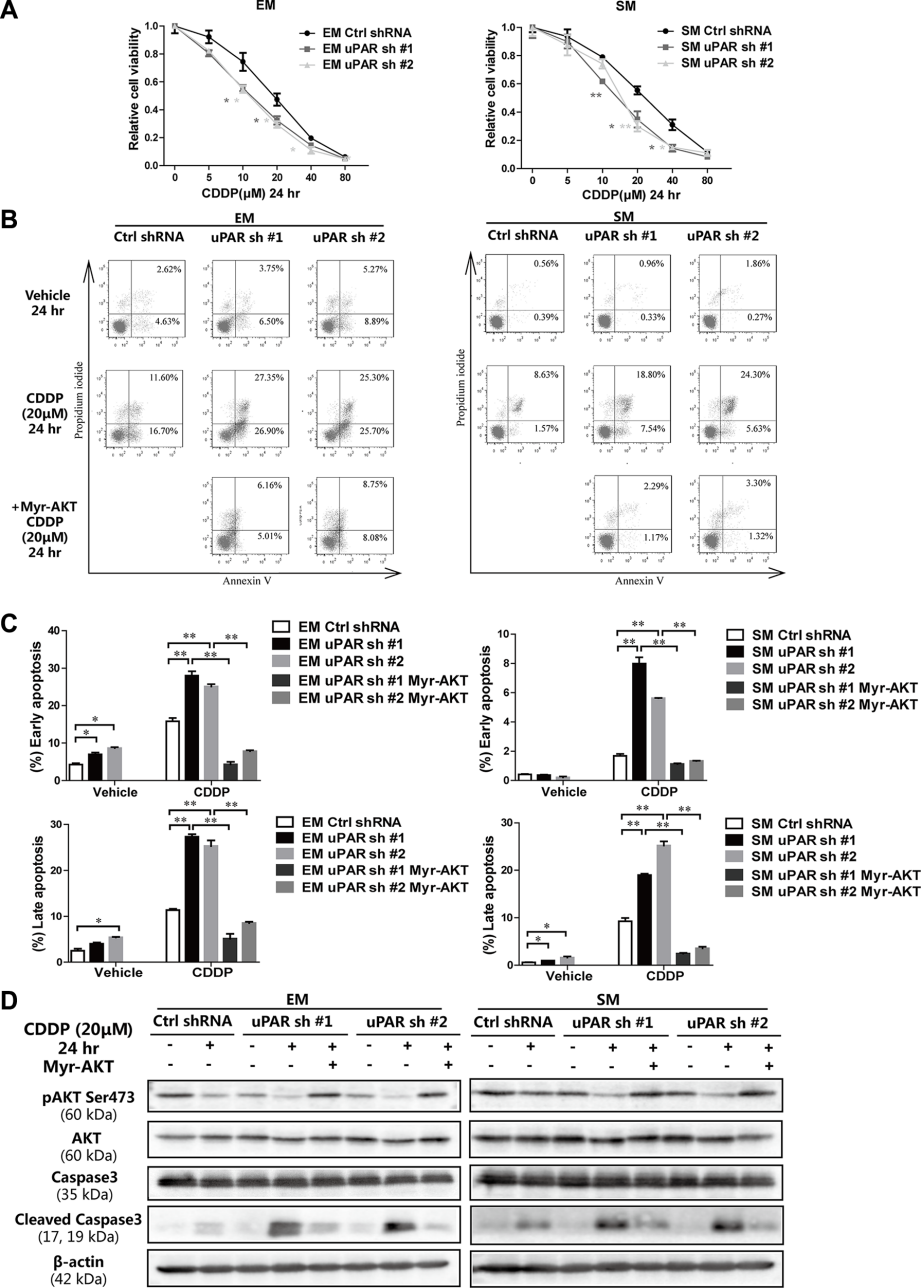
Increased cisplatin sensitivity leading to apoptosis after *uPAR* knockdown in rat MM cells (**A**) Relative cell viability by MTT assay 24 h after exposure to each concentration of cisplatin with *uPAR* knockdown in EM and SM cells. (**B**) Flow cytometry analysis and (**C**) its quantitation for increased early (AnnexinV^high^/PI^low^) and late (AnnexinV^high^/PI^high^) apoptosis with *uPAR* knockdown and rescued by myr-AKT transduction. (**D**) Caspase activation observed by cleaved caspase 3 in EM and SM cells via Western blot analysis after *uPAR* knockdown and rescued by myr-AKT transduction. EM, epithelioid subtype mesothelioma; SM, sarcomatoid subtype mesothelioma; CDDP, cisplatin (means ± SEM; *P < 0.05, **P < 0.01). See text for details.

To investigate the relationship between *uPAR* expression and chemotherapy sensitivity in MM, standard chemotherapeutic drugs for MM, pemetrexed (PTX) and cisplatin (CDDP) were evaluated in rat MM cells. Practically no difference in sensitivity to PTX was observed after *uPAR* knockdown (Supplementary Figure S4). In contrast, *uPAR* knockdown sensitized both EM and SM cells to CDDP at 24 h (Figure 7A). After CDDP treatment (20 μM at 24 h) with *uPAR* knockdown, the proportion of early/late apoptotic cells increased significantly, whereas *uPAR* knockdown alone did not induce significant MM cell death (Figure 7B, 7C). Because the AKT signaling pathway and its downstream proteins have been reported to interact with the caspase family to regulate cell apoptosis [23], we further evaluated the caspase-3 levels to determine whether decreased AKT activity induced by *uPAR* knockdown was associated with promoted apoptotic activity. Our results revealed that CDDP suppressed the *AKT* pathway, which was promoted by *uPAR* knockdown; this suppression led to activation of the apoptotic pathway, as shown by caspase-3 cleavage. Furthermore, we transducted a myristoylated form of AKT (myr-AKT) into *uPAR* knockdown cells to continuously express activated AKT (Supplementary Figure S5). The apoptotic activity was nearly abolished by sustained AKT activation (Figure 7D, Supplementary Figure S1J).

We also performed experiments with *uPAR* overexpression and CDDP treatment in Y-Meso-8A cells. We observed decreased sensitivity to CDDP after *uPAR* overexpression in Y-Meso-8A cells at 24 h (Supplementary Figure S6A). *uPAR* overexpression induced resistance to apoptosis after CDDP treatment, whereas a PI3K/AKT inhibitor, LY294002, and a specific AKT inhibitor, Akti-1/2, partially reversed the effect. These results indicate that resistance to apoptosis after CDDP treatment is partially related with AKT activity in MM cells (Supplementary Figure S6B, S6C).

### uPAR and AKT are simultaneously activated in human MM tissue

**Figure 8:**
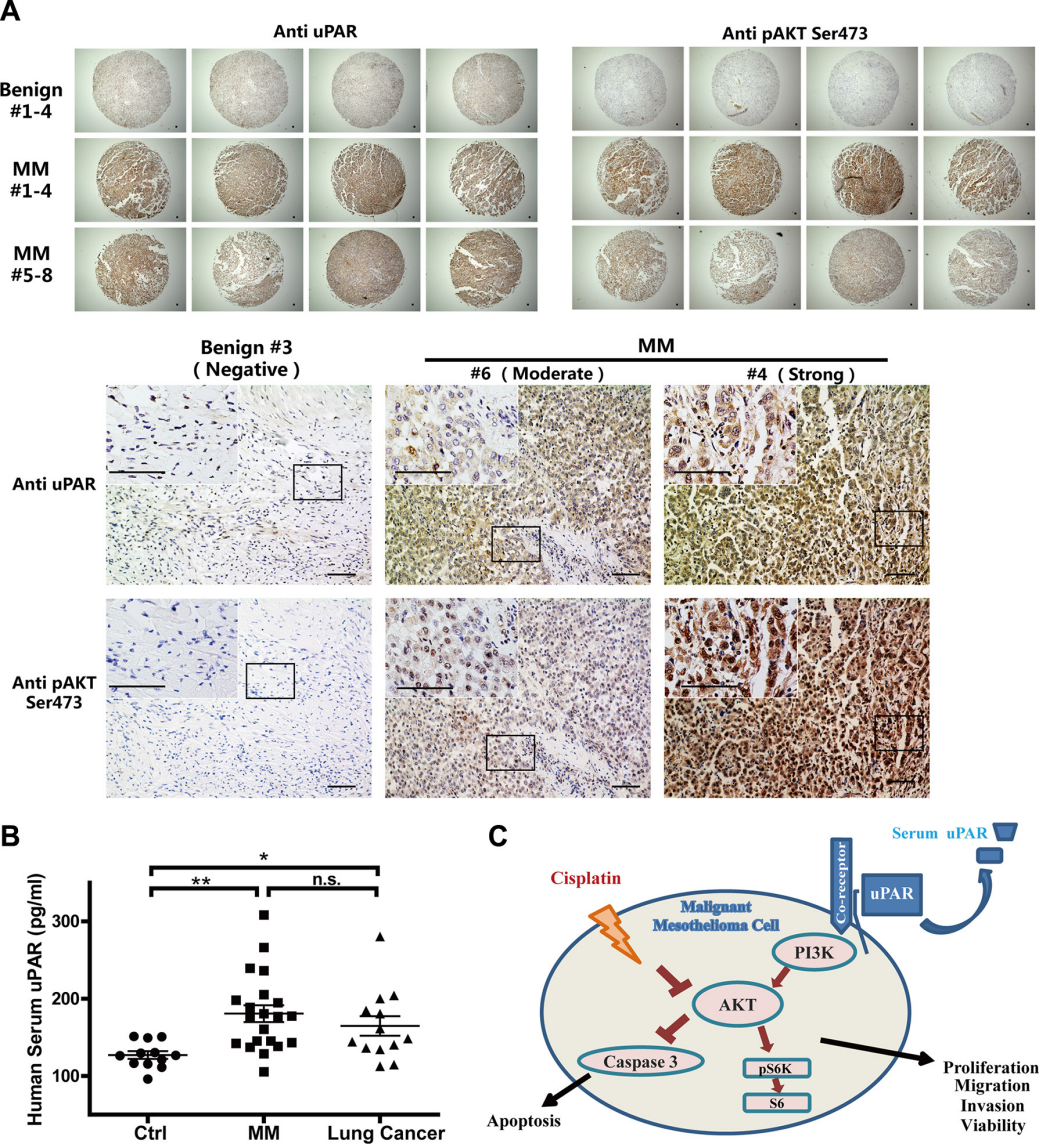
Increased uPAR levels in human MM tissues and in serum of MM patients (**A**) Immunohistochemical analysis of uPAR and pAKT in human MM tissue-array in comparison to benign pleural lesions (bar = 50 μm). (**B**) Increased serum uPAR levels in human MM and lung cancer patients groups, compared to the non-neoplastic control group (means ± SEM; *P < 0.05, **P < 0.01; m.s., not significant). (**C**) Summary scheme. uPAR-associated cellular signaling, and synergy of *uPAR* overexpression with reduced cisplatin sensitivity by activated AKT signalling in MM cells.

A human MM tissue array (T392a) from the Biomax company was subjected to immunochemical analysis for uPAR and activated AKT (pAKT, Ser473). Compared with benign pleural lesions, increased immunostaining of uPAR was observed in eight MM tissue cores from four patients, and pAKT showed corresponding alterations (Figure 8A).

### Elevated uPAR levels in the serum of MM patients

Because the soluble uPAR levels in cultured medium of MM cells was consistent with cellular expression (Supplementary Figure S7A, S7B), we further investigated the uPAR levels in the serum of MM patients. Based on the analyses of the control group, including 12 participants without malignant disease, versus the group of MM patients (*N* = 21; 15 EM and 6 BM) and 13 patients with lung cancer, we observed significantly increased serum uPAR levels in the MM patients. However, the lung cancer patients also exhibited high serum uPAR levels (Figure 8B). There were no significant differences between the MM and lung cancer patients.

## DISCUSSION

Recently, a comprehensive genomic analysis of MM identified recurrent mutations, gene fusions and splicing alterations [24]. Here, we used genome-wide expressing profiling from our animal carcinogenesis model to focus on *uPAR* [25, 26]. For the first time, we showed that *uPAR* overexpression is observed in asbestos-induced rat MM, regardless of the asbestos fibers used for carcinogenesis and the histological subtype of MM. Moreover, we found a significant correlation between survival and uPAR expressions in rat models. These data indicate that *uPAR* overexpression is a common and important expressional alteration in MM. However, we could not determine the relationship between *uPAR* expression and the histotype in human MM cells because of the lack of SM cell lines. Exposure to asbestos has been reported to upregulate *uPAR* expression in mesothelial cells [27]. Therefore, the link between this first reaction to asbestos and carcinogenesis highlighted the involvement of uPAR and it would be an interesting subject for future investigations.

Indeed, *uPAR* is overexpressed in some human cancers, including breast, gastric and lung cancer [28], and it has been associated with poor prognosis, particularly in cases of leukemia, lung, prostate and breast cancer [29–32]. Coincidently, *uPAR* overexpression was mostly reproduced in both rat and human MM cell lines, and with knockdown and overexpression studies *in vitro* and *in vivo*, we showed that *uPAR* is intimately associated with the malignant character of MM cells. However, it should be noticed that the proliferation and migration of MM cells can be increased to a relatively greater degree when stimulated with serum *in vitro* in addition to *uPAR* overexpression [19]. Also, some studies have reported that *uPAR* could activate cellular signaling and increase tumor cell malignancy in the absence of *uPA* through the binding of vitronectin [33, 34]. Our results confirmed that the suppressed effect of the proliferation and invasion with *uPAR* knockdown is not related to *uPA* expression in MM cells.

Although *uPAR* overexpression in human MM cell lines compared with MeT-5A and primary mesothelial cells has been previously reported [19, 35], we for the first time showed the association between *uPAR* overexpression and downstream PI3K/AKT/mTOR signaling with *uPAR* modulation in MM cells and xenograft tumors, as well as in human MM tissue samples. The results are consistent with those of a recent study in papillary thyroid carcinoma cells [36]. The PI3K/AKT/mTOR pathway is essential for cell proliferation and growth, cell cycle progression and cellular metabolism maintenance [37]. Furthermore, the PI3K/AKT pathway has been identified as a key regulator of survival during cellular stress [38]. Therefore, induced activation of AKT downstream genes by *uPAR* overexpression in MM can be considered a critical target for future MM treatment.

Many other signaling pathways have been reported to be associated with uPAR, including the focal adhesion kinase (FAK), EGFR, and Ras–mitogen-activated protein kinase (MAPK) pathways (epidermoid carcinoma) [22, 39], as well as the ERK pathway through formyl peptide receptor-like 1 (FPRL1; THP-1, macrophage), which is a G-protein-coupled receptor [40]. However, our results showed that ERK was not affected upon uPAR expression in MM cells which is similar to a report on nasopharyngeal carcinoma cells [41].

The identification of target molecules that confer chemoresistance in MM cells is of crucial importance for MM treatment. Our results showed that MM cells revealed caspase activation and apoptotic features with cisplatin treatment, which were inhibited by *uPAR* overexpression and promoted by *uPAR* knockdown. This is consistent with another relevant study identifying uPAR-positive cells that are resistant to cisplatin in small cell lung cancer [42]. We also found that the sensitivity of MM cells to cisplatin is related to *uPAR* induced AKT activation. To the best of our knowledge, this is the first report of *uPAR* expression being associated with cisplatin sensitivity through the AKT pathway in MM. As cisplatin is known to induce apoptotic cell death [43], several studies have reported on the role of the PI3K/AKT/mTOR signaling pathway in cisplatin sensitivity/apoptosis in different cell and cancer types (e.g., kidney cells and ovarian cancer) [44–46]. Different pathways are likely simultaneously activated by *uPAR* overexpression and may have crosstalk with each other [28]. Our results showed that PI3K/AKT or specific AKT inhibitors induced a slight increase in the apoptotic effect of cisplatin in *uPAR* overexpressing Y-Meso-8A cells, suggesting that other anti-apoptotic pathways are probably activated in MM cells.

Cell-surface uPAR can be shed from the membrane. The consistence of soluble uPAR levels in cultured medium and cellular expressions of MM cells with modulated uPAR expression were confirmed. Cleaved uPAR from the MM cell membrane leads to uPAR secretion into the blood flow, and our study was the first to show that MM patients have higher serum uPAR levels compared to those with benign diseases. The serum uPAR level is closely associated with its expression and has been suggested as a prognostic biomarker in some cancers (e.g., ovarian and prostate cancer) [47, 48]. Overexpression of *uPAR* downstream genes, such as *AKT* and *mTOR*, in MM tissues is associated with shortened MM patient survival [49]. With the ease of testing serum uPAR levels, our results suggest that the serum uPAR level could serve as a marker for MM diagnosis and therapy monitoring. However, as this marker is common to many different cancers, including lung cancer, analysis of the results requires ample caution.

In conclusion, we showed that elevated *uPAR* expression in MM increases AKT signaling activity, which is a major regulator of cisplatin-induced apoptosis (Figure 8C). Future studies on the interacting molecules, especially the coupling of *uPAR* expression with AKT activity in MM, are necessary to fully elucidate the molecular mechanisms. Antagonistic recombinant human antibodies against uPAR are currently available for putative diagnostic and therapeutic use in breast cancer [50]. The combination of uPAR abrogation and chemotherapeutic drugs in MM would be a promising therapeutic approach.

## MATERIALS AND METHODS

### Chemicals

*cis*-Diamminedichloroplatinum (cisplatin; CDDP; D3371) was obtained from Tokyo Chemical Industry, Japan. A PI3K inhibitor, LY294002 (129–04861), and vehicle control, dimethyl sulfoxide, were purchased from Wako, Japan. An AKT inhibitor, Akti-1/2 (A6730) was purchased from Sigma-Aldrich, USA.

### Asbestos-induced MM in rats and expression microarray analysis

Rat MM tissue samples confirmed by immunohistochemical analysis for mesothelioma markers from our previous experiments with this MM model were used [25]. Typical samples were randomly selected. Rat whole-genome microarrays (G4131F; Agilent Technologies, USA) were used; two normal mesothelial tissues obtained by scraping normal rat pleural and peritoneal cavities were used as controls [51], and 21 rat MM tissues (8 epithelioid subtype (EM) and 13 sarcomatoid subtype (SM)) were used as previously described [26] and registered as GEO Accession No. GSE48298 [26]. All expression data were normalized to β-actin. The animal experiment committee of Nagoya University Graduate School of Medicine approved this study.

### Cell lines, culture conditions and conditioned medium preparation

Rat MM cell lines (EM 1–5 and SM 1–5) were established from the ascites of mesothelioma-bearing rats models conducted in our previous studies [26]. Regarding 8 human MM cell lines, four cell lines, ACC-MESO-4, Y-MESO-8A, Y-MESO-9 and Y-MESO-25 were established in our laboratory (Y.S.) [52, 53]; NCI-H28 and MSTO-211H were purchased from ATCC (USA), and NCI-H290 and NCI-2052 were kindly provided from Dr. Adi F. Gazdar. Rat peritoneal mesothelial cells (RPMCs) were cultured from the omentum of Wistar rats, and full-length HPV16E6 and E7 were transfected for immortalization as previously described [54]. RPMC and rat/human mesothelioma cell lines were all maintained and used for experiments (except for conditioned medium preparation) in 1640 medium supplemented with 10% FBS and 1% antibiotic-antimycotic solution in a 5% CO_2_ incubator at 37°C. A line of immortalized mesothelial cells, MeT-5A, was purchased from ATCC and cultured in M199, according to the instructions. To prepare conditioned medium, subconfluent cultures of 1 × 10^6^ cells were washed twice with PBS and incubated with serum-free medium for 24 h. Conditioned medium was collected and filtered through a 0.22 μm filter, the same amount of which was loaded for SDS-PAGE as described [26].

### Tissue array, immunohistochemistry and quantitative analysis

A rat MM tissue array, including 20 samples and 3 control tissues, was prepared in our laboratory. A human mesothelioma tissue array was purchased (Biomax, USA). The avidin-biotin complex method with peroxidase or immunofluorescence was used for the immunohistochemical analysis, as previously described [55, 56]. All images were obtained using a BZ-9000 microscope (Keyence, Japan). The uPAR protein levels in the rat tissue arrays were assessed based on the H-score [57] in a blinded manner by S.W. and Y.H. The H-score was calculated as the sum of the percentage of stained cells, multiplied by an ordinal value corresponding to the intensity level (0 = none, 1+ = weak, 2+ = moderate, 3+ = strong), according to the following formula: 1 × (% cells 1+) + 2 × (% cells 2+) + 3 × (%cells 3+). The 4 intensity levels resulted in the following score ranges: 0–300; mild (0–100), moderate (101–200) and strong (201–300). Survival curves for the mesothelioma rats were plotted using the Kaplan-Meier method according to an H-score threshold of 200, and the differences were compared using the log-rank test.

### Tumor xenografts experiments

BALB/c nu/nu mice (4–6 weeks old; SLC, Japan) were subcutaneously injected into the flanks with 100 μl of PBS containing 5 × 10^6^ MM cells. Tumor development was monitored everyweek by measuring the tumor volume determined by the following formula: (Length × Width^2^)/2. The tumors were harvested after euthanization. All procedures were performed in accordance with the national guidelines and approved by the animal experiment committee of Nagoya University Graduate School of Medicine.

### Antibodies

The following antibodies were used: anti-uPAR (9692; Western blot analysis), anti-pAKT Ser 473 (4051), anti-AKT (4691), anti-p-pS6K Thr 389 (9205), anti-pS6K (9202), anti-pS6 Ser 235/236 (4857), anti-S6 (2217), anti-caspase-3 (9665), anti-cleaved caspase-3 (9664) from Cell Signaling Technology, USA; anti-uPAR (103791; immunohistochemistry) from Abcam, USA; anti-uPA (sc-14019) from Santa Cruz, USA; anti-HA Tag (04–902) from Millipore, USA and anti-β actin (A1978) from Sigma-Aldrich, USA.

### Annexin V-FITC/PI flow cytometric analysis

The APOAF Annexin V apoptosis kit (Sigma, USA) was used for staining annexin V on the outside of the apoptotic cells according to the manufacturer's protocol. All samples were quantified using a Canto II flow cytometer (BD Biosciences, USA) and analyzed by FlowJo software 7.6 software (TreeStar, USA). AnnexinV^high^/PI^low^ cells were recognized as early apoptosis and AnnexinV^high^/PI^high^ cells were identified as late apoptosis or necrosis.

### Determination of serum uPAR concentration in humans

A human uPAR Quantikine ELISA Kit (DUP0012, R&D Systems, USA) was used with the human serum samples according to the manufacturer's protocol. All subjects provided written informed consent. The study was conducted in accordance with the ethical standards, the Declaration of Helsinki and national and international guidelines, and it was approved by the Ethical Committee of the Nagoya University Graduate School of Medicine.

### Statistical analysis

The data were analyzed using PASW 22.0 (SPSS, USA) and GraphPad Prism 5 (GraphPad Software, USA). Statistical significance between two groups of interest was analyzed using the unpaired Student's *t-test*. One-way analysis of variance (ANOVA) and the least significant difference (LSD) test were used to analyze more than two subgroups. The results are shown as the mean ± SEM except for where noted. A *P* value of < 0.05 was considered significant.

## SUPPLEMENTARY MATERIALS



## Abbreviations

BM: biphasic subtype mesothelioma
CDDP: cis-diamminedichloroplatinum
DAPI: 2-(4-amidinophenyl)-1H-indole-6-carboxamidine
DMSO: dimethysulfoxide
EM: epithelioid subtype mesothelioma
ERK: extracellular signal-regulated kinase
GPI: glycosyl-phosphatidylinositol
MM: malignant mesothelioma
mTOR: mammalian target of rapamycin
MTT: 3-(45-di-methylthiazolyl-2-yl)-25-diphenyltetrazolium bromide
PBS: phosphate-buffered saline
PI3K: phosphatidyl inositide 3-kinase
PTX: pemetrexed
RPMC: rat peritoneal mesothelial cell
SDS-PAGE: sodium dodecyl sulfate-polyacrylamide gel electrophoresis
SM: sarcomatoid subtype mesothelioma
uPA: urokinase-type plasminogen activator
uPAR: urokinase-type plasminogen activator receptor.
