# Supplementary material for: Urokinase-type plasminogen activator receptor promotes proliferation and invasion with reduced cisplatin sensitivity in malignant mesothelioma
Source: Oncotarget. 2016 Sep 2;7(43):69565–78. doi: 10.18632/oncotarget.11829 (PMC5342498; doi:10.18632/oncotarget.11829)
Supplement: Supplementary file 1 [file oncotarget-07-69565-s001.pdf]

Urokinase-type plasminogen activator receptor promotes proliferation and invasion with reduced cisplatin sensitivity in malignant mesothelioma

Supplementary Materials

SUPPLEMENTARY METHODS

Immunofluorescence

Cells were grown on chamber slides and were fixed with 4% formaldehyde and incubated with 2% goat serum in PBS at room temperature for 1 h for blocking, followed by incubation with primary antibody (1:200) at 4°C overnight. After washing, cells were incubated with Alexa488-labeled secondary antibody (1:500) (Biotium, USA) at room temperature for 1 h. The slides were washed three times, covered with glass cover slips using a DAPI-containing mounting media, followed by image analysis with Zeiss LSM 710 confocal microscope (Zeiss, Germany).

Western blot analysis

Cell lysates were prepared by ice-cold Lysis-M buffer (Roche, Switzerland) supplemented with cOmplete protease inhibitor and PhosSTOP phosphatase inhibitor tablets (Roche, Switzerland). After centrifuge at 14,000 × g for 10 min, protein concentration of the supernatants were determined with BCA assay (Nacalai Tesque, Japan). Cell extracts were subjected to SDS-PAGE, and proteins were transferred to PVDF membranes (Merck Millipore, Germany) and probed with primary antibodies (1:1000). Subsequently, they were probed with HRP-conjugated secondary antibodies (1:2000), and signals were detected using ECL reagents (GE Healthcare, USA). Densitometric analysis was peformed using the ImageJ software (NIH, USA).

Primer sequences for semiquantitative RT-PCR analysis

Semiquantitative RT-PCR was performed using Platinum SYBR green master mix kit (Invitrogen, USA) on ABI 7300 Real-Time PCR System (Applied Biosystems, USA) according to the manufacturer's protocol. Gene expression is presented as a ratio of the housekeeping gene GAPDH expression. The primer used for rat *uPAR (Plaur)* was 5′-GGACGAACCCTACACCAAAG-3′ and 5′-CATTGCATTGGGTGAAGTTG-3′, for human *uPAR (PLAUR)* was 5′-GACCCTGAGCTATCGGACT-3′ and 5′-GCATTCGAGGTAACGGCTTC-3′, for rat *uPA (Plau)* was 5′-ACAATGCTCACAGATCCGATG-3′ and 5′-AGGCCAATTTGCACATAGCAC-3′, for human uPA (PLAU) was 5′-AGGCTTAACTCCAACACGCAAG-3′ and 5′-CTTCAGCAAGGCAATGTCGTT-3′.

Lentiviral short hairpin RNA expression vectors

Short hairpin RNAs (*sh*RNA) targeting rat *uPAR* as 5′-gatccGTTCAGAGCTTACCACCGAATGTTCAAGA GACATTCGGTGGTAAGCTCTGAATTTTTTg-3′, 5′-gat ccGTGCTGGGAAACCGGAGTTACTTCAAGAGAGT AAC TCCGGTTTCCCAGCACTTTTTTg-3′ and targeting rat uPA as 5′-gatccGAGGAAAG GCCAATACTGATTC AAGAGATCAGTATTGGCCTTTCCTCTTTTTTg-3′, 5′-gatccG CGGGAGATTCAGGAGGACCTCTTATTTCAA GAGAATAAGAGGTCCTCCTGAATCTCCCGTTTTTT g-3′ and a control *sh*RNA targeting the luciferase gene as 5′-gatccGTGCGTTGTTAGTACTAATCCTATTTTTC AAGAGAAAATAGGGTTGGTACTAGCAACGCACTT TTTTg-3′ were cloned into *sh*RNA expression vectors pSIF-H1 (System Biosciences, USA). Lentiviruses were generated in HEK293T cells by vira-power lentiviral packaging mix (Invitrogen, USA). Infected rat cells were selected with puromycin at 1–2 mg/mL at least for two weeks.

Expression vector

Total length of human *uPAR* cDNA (NM_002659) was cloned into pcDNA 3.1 vector (Invitrogen, USA). A pcDNA3 Myr HA Akt1 plasmid was obtained from Addgene, USA. Cells were transfected by Fugene HD (Promega, USA) and transfected cells were selected with G418 (Wako, Japan) at 800–3000 mg/ml at least for 2 weeks.

MTT assay

Same amount of cells suspension (100 µl) was added to a 96-well plate. Following incubation and treatment, 10 µl MTT stock solution (5 mg/ml in PBS; Sigma-Aldrich, USA) was added to each well. Cells were subsequently incubated at 37°C for 3 h, then supernatant was discarded and 100 µl dimethyl sulfoxide (Wako, Japan) was added and the plate was agitated in the dark. Finally, the optical density (OD) was detected using a microplate reader (Powerscan4; Bio-Tek Instruments, USA) at a wavelength of 540 nm with a reference wavelength of 630 nm.

Cell counting assay

Same amount of cells was plated to a 12-well plate. After different time of incubation, cells from each well were collected. Cell suspension was counted by trypan blue exclusion method by a standard hemocytometer counting chamber (ERMA, Japan).

Migration and invasion assay

Cell migration and invasion assays were performed using the transwell permeable supports (Boyden chamber inserts) with 8 mm pore filters uncoated (for migration) and coated with matrigel (for invasion) according to the manufacturer's protocol (BD Biosciences, USA). Cells were added to the upper chambers in medium with 1% FBS, and medium with 10% FBS was added to the lower chambers. After 24 h-incubation at 37°C, the cells that had not migrated were removed from the upper surface of the filters with cotton swabs. The cells that had migrated to the lower surface of the filters were fixed in 4% paraformaldehyde and stained with 0.2% crystal violet. Migration and invasion were quantified by counting cells in seven randomly selected fields on each filter under a microscope at 100 × magnification.

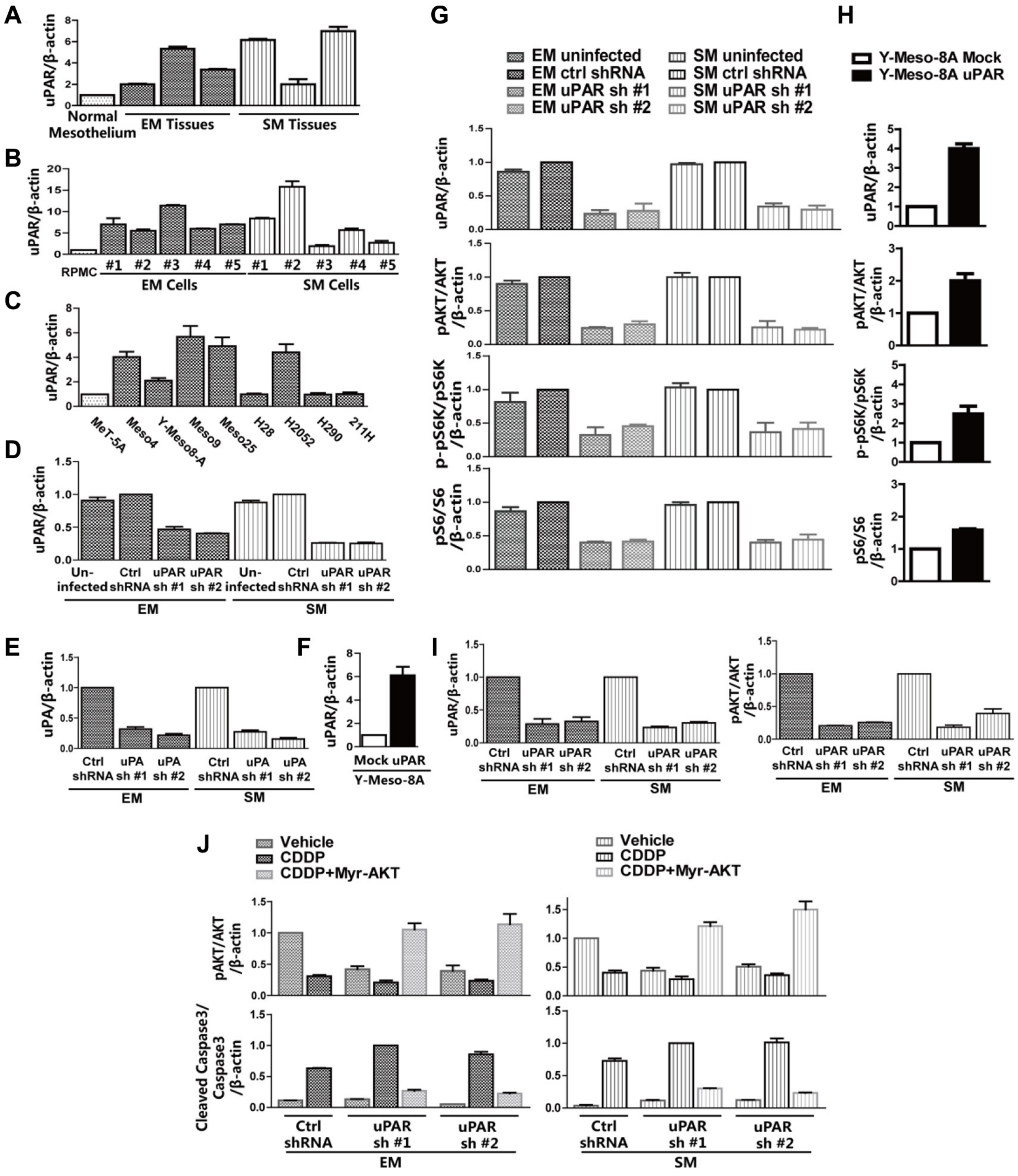

Supplementary Figure S1: Quantitation of bands in Western blot analyses by densitometry. Quantitation of bands in Western blot analyses shown in (**A**) Figure 1C (**B**) Figure 1F, (**C**) Figure 1G, (**D**) Figure 2B, (**E**) Figure 3E, (**F**) Figure 4A, (**G**) Figure 5A, (**H**) Figure 5B, (**I**) Figure 6C and (**J**) Figure 7D. EM, epithelioid subtype mesothelioma; SM, sarcomatoid subtype mesothelioma; RPMC, rat peritoneal mesothelial cell; Myr-AKT, myristoylated AKT; CDDP, cis-diamminedichloroplatinum.

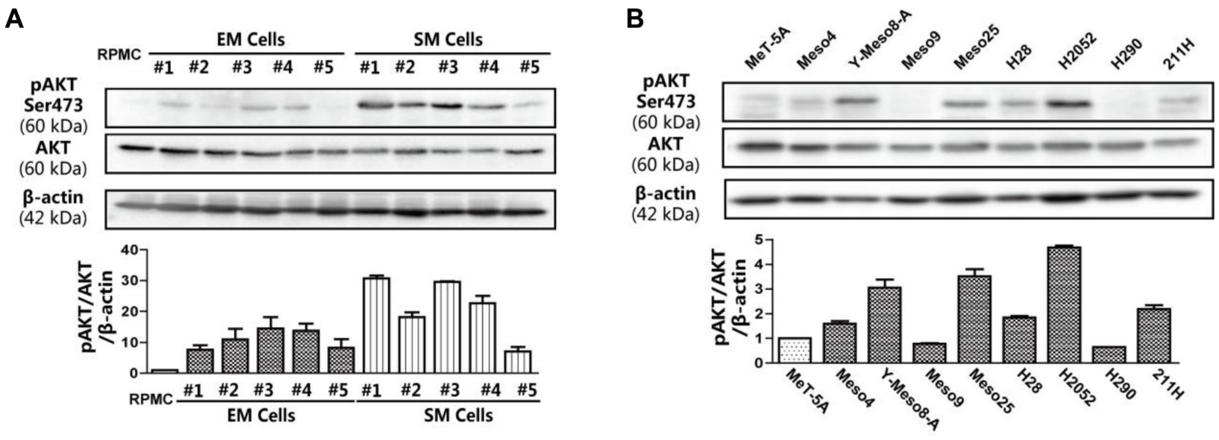

Supplementary Figure S2: AKT activation in rat/human MM cells. Most of (**A**) rat and (**B**) human MM cells showed activation of AKT compared with control cells by Western blot and densitometric analyses. EM, epithelioid subtype mesothelioma; SM, sarcomatoid subtype mesothelioma; RPMC, rat peritoneal mesothelial cell.

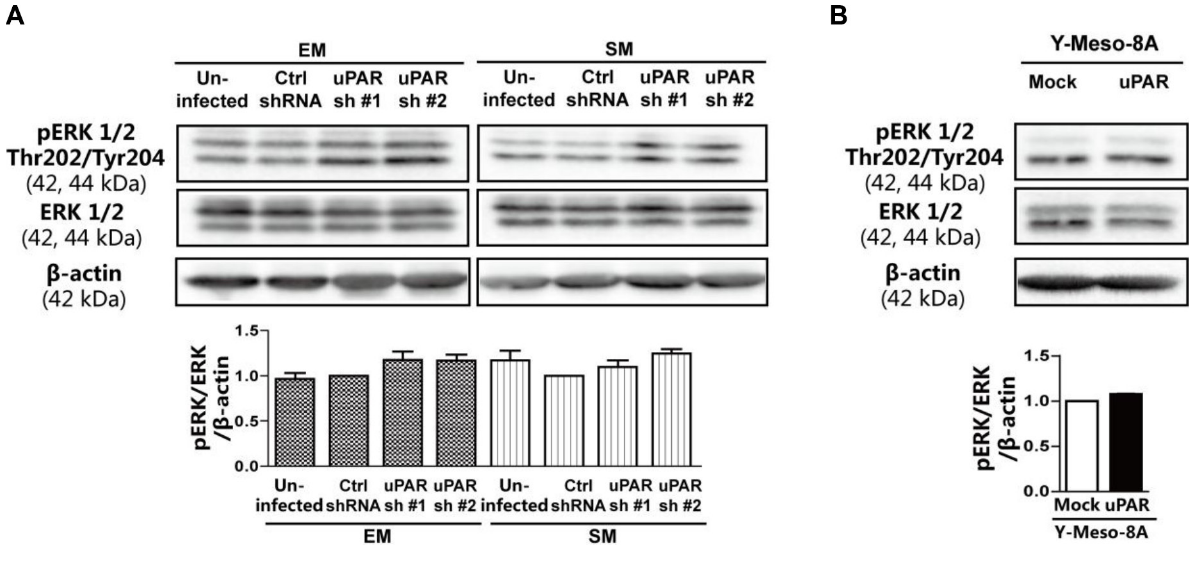

Supplementary Figure S3: Neither *uPAR* knockdown in rat MM cells nor *uPAR* overexpression in human MM cells has effect on ERK expression. (**A**) Downregulated *uPAR* expression in EM and SM cells and (**B**) overexpression of *uPAR* in Y-Meso-8A showed no effect on ERK expression by Western blot and densitometric analyses. EM, epithelioid subtype mesothelioma; SM, sarcomatoid subtype mesothelioma.

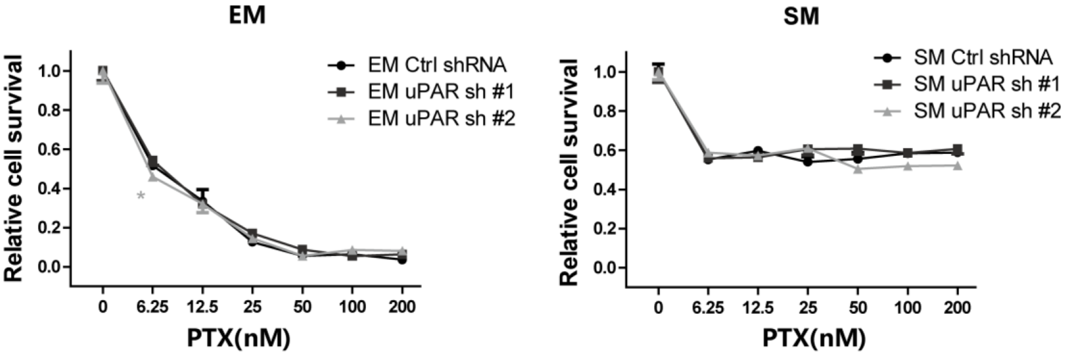

Supplementary Figure S4: Pemetrexed treatment in rat MM cells with *uPAR* knockdown. Downregulated *uPAR* expression in EM and SM cells showed no obvious difference to pemetrexed treatment, compared with control cells by MTT assay. EM, epithelioid subtype mesothelioma; SM, sarcomatoid subtype mesothelioma; PTX, pemetrexed (means ± SEM; *$P < 0.05$).

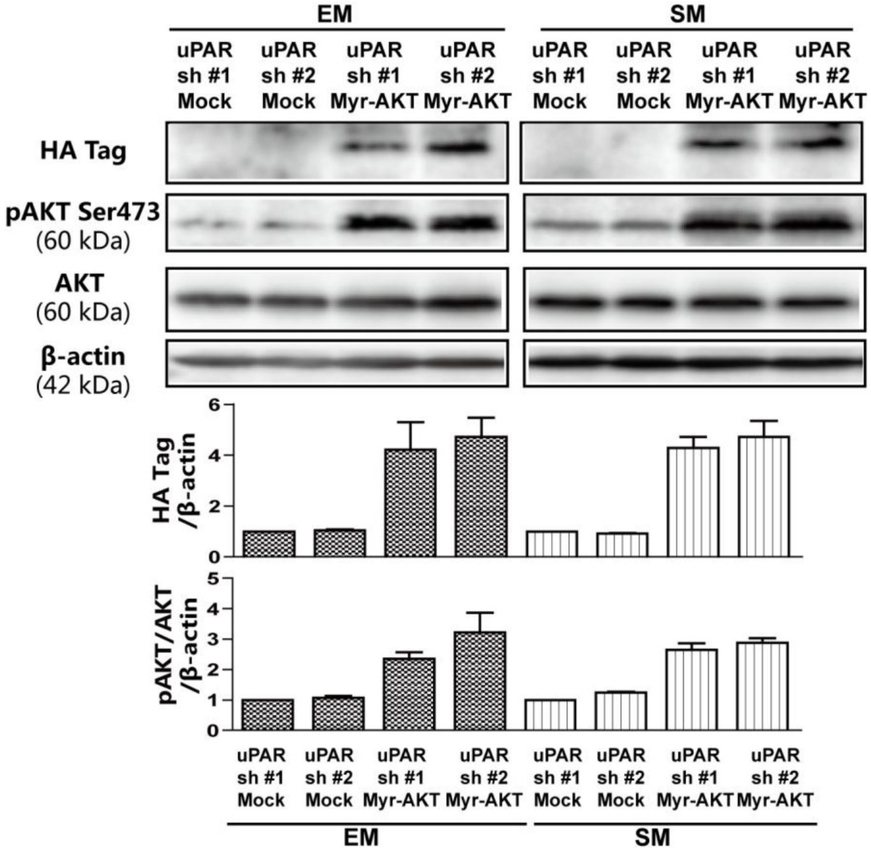

Supplementary Figure S5: Transduction of myristoylated form of AKT in rat MM cells with *uPAR* knockdown. Transduction of a continuously active form of AKT by myr-AKT-HA plasmid in EM and SM cells determined by Western blot and densitometric analyses. EM, epithelioid subtype mesothelioma; SM, sarcomatoid subtype mesothelioma.

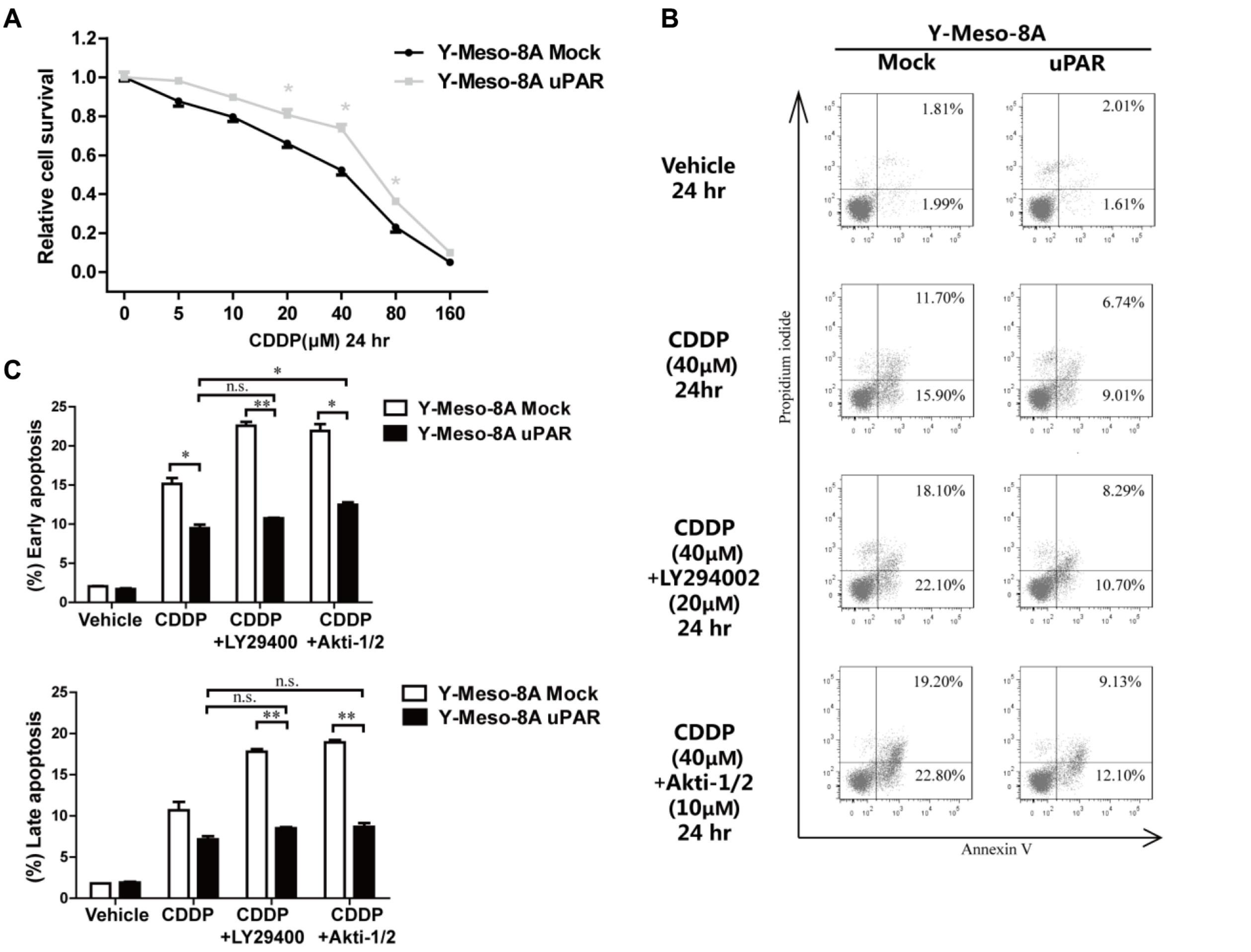

Supplementary Figure S6: Decreased cisplatin sensitivity avoiding apoptosis after *uPAR* overexpression in Y-Meso-8A cell through *AKT* activation. (**A**) Relative cell viability by MTT assay 24 h after exposure to each concentration of cisplatin under *uPAR* overexpression in Y-Meso-8A cells. (**B**) Flow cytometry analysis and (**C**) its quantitation for increased early (AnnexinV^{high}/PI^{low}) and late (AnnexinV^{high}/PI^{high}) apoptosis (means ± SEM; *$P < 0.05$, **$P < 0.01$).

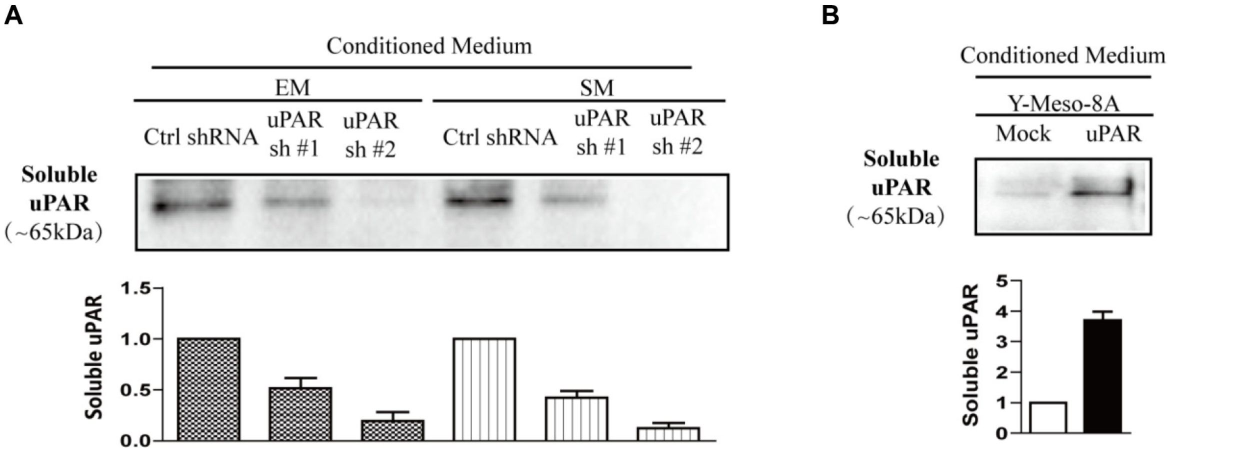

Supplementary Figure S7: Soluble uPAR in conditioned medium from MM cells with *uPAR* knockdown or overexpression. (**A**) Downregulated *uPAR* expression in EM and SM cells showed decreased soluble uPAR in conditioned medium compared with control cells by Western blot and densitometric analyses. (**B**) Overexpression of *uPAR* in Y-Meso-8A cell showed increased soluble uPAR in conditioned medium Western blot and densitometric analyses. EM, epithelioid subtype mesothelioma; SM, sarcomatoid subtype mesothelioma.
